# Safety and Cost-effectiveness of LigaSure® in Total Thyroidectomy in Comparison with Conventional Suture Tie Technique

**DOI:** 10.7759/cureus.6368

**Published:** 2019-12-12

**Authors:** Mehreen K Bhettani, Mubarik Rehman, Muhammad S Khan, Humera N Altaf, Kamran Hakeem Khan, Fareeha Farooqui, Mohammad Amir, Omar S Altaf

**Affiliations:** 1 General Surgery, Royal Oldham Hospital, Manchester, GBR; 2 General Surgery, Shifa College of Medicine, Islamabad, PAK; 3 General Surgery, Shifa Tameer E' Millat University, Shifa International Hospital, Islamabad, PAK; 4 Surgery, Shifa International Hospital, Islamabad, PAK; 5 General Surgery, Nowshera Medical College, Nowshera, PAK; 6 Surgery, Shifa College of Medicine, Islamabad, PAK; 7 General Surgery, Shifa College of Medicine, Shifa International Hospital, Islamabad, PAK; 8 General Surgery, Tehsil Headquarter Hospital, Attock, PAK

**Keywords:** thyroid, total thyroidectomy, complications, haemorrhage, hypocalcemia, parathyroid

## Abstract

Introduction: Total thyroidectomy has been considered to be a treatment of choice for thyroid malignancies. It has recently gained popularity as gold standard for benign thyroid disorders requiring surgical treatment. Because of higher number of complications associated with total thyroidectomy, it is still considered an adventurous endeavor. Most important improvements in thyroid surgery include laparoscopic thyroidectomy, energy based devices (EBD) like Harmonic Focus® and LigaSure® for dissection and hemostasis, intraoperative neuromonitoring, and parathyroid hormone (PTH) assay technology.

Aim of Study: Recent studies have suggested that despite lesser complication rates with LigaSure^ ^dissection method in total thyroidectomy, it is associated with prolonged operation time. Aim of our study was to compare conventional suture tie technique and LigaSure thyroidectomy, in terms of perioperative complications including bleeding, recurrent laryngeal nerve (RLN) injury, postoperative hematoma, hypocalcemia, operation time, and cost-effectiveness.

Results: This is a retrospective descriptive study done at Shifa Internationall Hospital/Shifa College of Medicine/Shifa Tameer e’ Millat University, Islamabad, Pakistan from 1st of June 2016 to 1st of June 2018. One hundred and two (102) patients who underwent surgical treatment for benign thyroid diseases were included in the study. Study was done after approval from ethical committee of Shifa International Hospital/Shifa Tameer e’ Millat University. Group A included patients in whom LigaSure was used for hemostasis and dissection during total thyroidectomy. Group B included patients who underwent total thyroidectomy by traditional clamp, tie, and electrocautery method for hemostasis and dissection. Frequency of female patients in group A was 45 (88.2%) and in group B was 41 (80.3%). In group A frequency of male patients was 9 (17.6%) and in group B it was 7 (13.7%). Mean operative time in group A was significantly lower (92 ± 9.61 min) than group B (123 ± 7.96 min). Mean intraoperative blood loss in group A was estimated to be 51.73 ± 5.65 mL and 139.42 ± 7.31 mL in group B. Transient hypocalcemia was the most common complication in both the groups. In group A 6.8% (n=7) patients developed transient postoperative hypocalcemia. Three patients in group B (2.9%) had transient hypocalcemia.

Conclusion: LigaSure was significantly advantageous over conventional technique in reducing risk of complications and operation time as well as perioperative and postoperative blood loss. The reduction of operative times resulted in decreased operating room occupancy costs but the overall cost of surgery was significantly higher in LigaSure group.

## Introduction

Total thyroidectomy has been considered to be a treatment of choice for thyroid malignancies. It has recently gained popularity as gold standard for benign thyroid disorders requiring surgical treatment [1]. According to American Thyroid Association approximately 150,000 patients undergo thyroidectomy every year in the United States for benign or malignant disease [2]. Transient hypocalcemia and recurrent laryngeal nerve (RLN) injury are the most commonly reported complications (5%-15%) [2-3]. Because of higher complication rate associated with total thyroidectomy, it is still considered an adventurous endeavor [3]. Most important improvements in thyroid surgery include laparoscopic thyroidectomy, energy based devices (EBD) like Harmonic Focus® (Ethicon by Johnson & Johnson) and LigaSure® (Medtronics) for dissection and hemostasis, intraoperative neuromonitoring, and parathyroid hormone (PTH) assay technology [4-5]. Better hemostasis allows for early removal of drain and decreased hospital stay [6-7]. Some authors do consider the increased healthcare costs associated with use of EBDs to be somewhat of a disadvantage [8]. Most importantly EBDs are indispensable in almost all endoscopic procedures for cutting and hemostasis without increasing the risk of postoperative complications [9-11]. 

Recent studies have suggested that despite advantage of lesser complication rates LigaSure dissection method in total thyroidectomy is associated with prolonged operation time [3, 7-12]. In our study our aim was to compare conventional suture tie technique and LigaSure thyroidectomy, in terms of perioperative complications including bleeding, RLN injury, postoperative hematoma, hypocalcemia, operation time, and cost-effectiveness.

## Materials and methods

This is a retrospective descriptive study done at Shifa International Hospital/Shifa College of Medicine/Shifa Tameer e’ Millat University Islamabad, Pakistan from 1st of June 2016 to 1st of June 2018. One hundred and two patients who underwent surgical treatment for benign thyroid diseases were included in the study. Study was done after approval from ethical committee of Shifa International Hospital/Shifa Tameer e’ Millat University.

All patients who underwent total thyroidectomy for toxic or simple multinodular goiter, diffuse goiter, Grave’s disease, retrosternal goiter, and other indications for thyroid surgeries were included in the study. Patients who underwent thyroidectomy for thyroid cancers or had central or lateral neck dissection for lymphadenopathy, recurrent goiter, ablation with radioiodine were excluded from the study.

Hospital charts of patients were reviewed for data collection for this research. Slovin’s formula n = N / (1+Ne2) n = no. of samples N = total population, e = error margin/margin of error [3]. Sampling technique was nonprobability convenience sampling. Patients fulfilling the inclusion criteria were contacted via phone to obtain consent for inclusion in the study. Age, gender, thyroid disease, preoperative serum calcium levels, thyroid function tests, chest X-rays, electrocardiogram (ECG), and indirect laryngoscopy (IDL) were documented.

Patients were divided into two groups. Group A included patients in whom LigaSure was used for hemostasis and dissection during total thyroidectomy. Group B included patients who underwent total thyroidectomy by traditional clamp, tie, and electrocautery method for hemostasis and dissection. Superior pole of thyroid gland was ligated by using Vicryl 1/0 suture and inferior thyroid artery was ligated using Vicryl 2/0 suture. All patients were given due respect and their comfort was taken care of during the study. Drain output was noted from postoperative charts and documented in our performa. Postop complications were documented.

The data were analyzed using SPSS version 20. Descriptive statistics was used to calculate mean, standard deviation for numerical variables like age, duration of admission, duration of surgery, cost of procedure, and serum calcium levels. While frequency and percentages were presented for categorical variables like clinical diagnosis, history of premedication for thyroid disease, tissue diagnosis, isotope scan, IDL, operative findings, procedure adopted, status of recurrent laryngeal nerve (RLN), vocal cords at extubation, primary hemorrhage, tension hematoma, preservation of parathyroid and their blood supply, wound hematoma, postop pain, wound seroma, hoarseness of voice, and hypocalcemia. Operative procedure and findings were documented in performa attached.

The statistical analysis to compare outcomes between two groups was done using central trend measurements, Student's t, chi-square, and Fisher's exact test, with a significance level of p < 0.05. The cost-effectiveness analysis was completed by determining the total cost of the surgery including cost of operative time and hospital stay. Comparison of efficacy was evaluated from postoperative complications. 

## Results

The total number of patients included in the study was 102 (n=102). Number of female patients was 45 (88.2%) and 41 (80.3%) in groups A and B, respectively. In group A frequency of male patients was 9 (17.6%) and in group B it was 7 (13.7%). The mean age of the patients undergoing surgery was 41.7 ± 7.1 (group A) and 39.4 ± 6.7 (group B); minimum age was 18 years, and maximum age was 67 years (Table 1).

**Table 1:** Patients’ demographics.

Variable	LigaSure® (n ¼ 51) Group A	Conventional suture tie technique (n ¼ 51) Group B
Age	41.3 ± 7.1	39.4 ± 6.7
Male	9 (17.6%)	7 (13.7%)
Female	45 (88.2%)	41 (80.3%)

A total of 21 patients (20.5%) needed treatment with anti-thyroid drugs and beta blockers to be rendered euthyroid before surgery. Sixty eight patients (66.6%) had simple multinodular goiter (clinically as well as biochemically). There was only one patient (0.9%) who had been diagnosed with differentiated thyroid cancer preoperatively (Table 2).

**Table 2:** Thyroid disorders in study population.

Variable	LigaSure® technique (n = 51) Group A	Conventional suture tie technique (n = 51) Group B	Percentage
Retrosternal multinodular goiter	1	2	2.9%
Toxic multinodular goiter (euthyroid with drugs)	11	10	20.5%
Simple multinodular goiter	33	35	66.6%
Grave’s disease	5	4	8.8%
Differentiated thyroid cancer	1	0	0.9%

**Table 3:** Comparison of perioperative parameters between LigaSure® and conventional suture tie technique.

Peri-operative parameters	LigaSure® technique (n ¼ 51) Group A	Conventional suture tie technique (n ¼ 51) Group B	<>
Patients	51	51	
Operative time (min)	92 ± 9.6	123 ± 7.9	<0.01
Per-op blood loss (mL)	51.73 ± 5.65	139.42 ± 7.31	<0.01
Postoperative calcium (mg/dL)	8.62 ± 2.1	7.55 ± 1.6	<0.05
Drainage volume (mls)	33.5 ± 4.13	73.8 ± 5.71	<0.05
Hospital stay (days)	1.8 ± 0.7	3.2 ± 0.6	<0.05
Cost of surgery PKR	190,000	138,000	<0.05
Re-operation	0	1	>0.05
Mortality	0	0	0

As shown in Table 3 mean operative time in group A was significantly lower (92 ± 9.61 min) than group B (123 ± 7.96 min). The difference in the mean operating time between the two groups was statistically significant (p<0.01). Mean intraoperative blood loss in group A was estimated to be 51.73 ± 5.65 mL and 139.42 ± 7.31 mL in group B. The difference between the two groups was statistically significant (p<0.01). Similarly the postoperative blood loss between the two groups was also found to be statistically significant (p<0.01). Mean drain output was 33.50 ± 4.13 mL in group A and 73.80 ± 5.71 mL in group B. There was one case of hematoma formation in the conventional vascular ligature group (0.9%) that required reoperation while none of the patients undergoing total thyroidectomy with LigaSure developed hematoma formation. The difference between the two groups was statistically insignificant (p>0.05). None of the patients in both the groups developed surgical site infection. The results have been summarized as shown in Table 4.

**Table 4:** Postoperative complications. RLN, recurrent laryngeal nerve

Complications	LigaSure® n= 51 Group A	Conventional technique n= 51 Group B	< >
Neck hematoma	0	0.9% (1)	>0.05
Transient hypocalcemia	2.9% (3)	6.8% (7)	<0.05
Transient RLN palsy	0.9% (1)	1.9% (2)	>0.05
Permanent hypoparathyroidism	0.9% (1)	3.9% (4)	<0.05
Permanent RLN palsy	0	0	>0.05
Wound infection	0	0.9% (1)	>0.05
Death	0	0	>0.05
Overall	4.9% (5)	14.7% (15)	<0.05

There was no mortality in either group. Transient hypocalcemia was the most common complication in both the groups. In group A 6.8% (n=7) patients developed transient postopertative hypocalcemia. Three patients in group B (2.9%) had transient hypocalcemia. The difference between the two groups was statistically significant (p<0.05). RLN was identified dissected along its entire course and preserved in all surgeries. Hoarseness of voice was present in two patients (1.9%) in group B and in one patient in group A. All patients had transient RLN injury. Their hoarseness of voice had recovered between three and six months and their IDL showed normal vocal cord movement. 

## Discussion

In order to avoid risk of complications in total thyroidectomy, emphasis is given to thorough dissection for precise identification of anatomical structures and accurate hemostasis [12]. The most common complications of total thyroidectomy are injury to RLN, external branch of superior laryngeal nerve, parathyroid gland, esophagus and trachea and hemorrhage from thyroid vasculature [13].

Molnar et al. reported their observation that using the LigaSure small jaw device to perform total thyroidectomy is a reliable option [13]. Sutureless thyroid surgery has gained grounds in the last decade [14]. In recent studies effectiveness of LigaSure in hemostasis and dissection has been demonstrated in thyroidectomies. However, the effect of LigaSure use in reducing operation time, hospital stay, and intraoperative and postoperative complications are controversial [15].

The effectiveness and safety of EBDs like Harmonic-Scalpel® (Ethicon by Johnson & Johnson) and LigaSure have been proven in many studies [7, 10, 16]. In our study population number of females was greater than males. Our study included 45 (88.2%) female patients in group A and 41 (80.3%) in group B. In group A frequency of male patients was 9 (17.6%) and in group B it was 7 (13.7%). Similar to our study Ahmed et al. in their study at Pakistan Emirates Military Hospital included 66 female patients (94.3%) and only four male patients (5.7%) [17]. The mean age of patients in our study was 39.4 ± 6.7 years in conventional suture ligation technique group, and it was 41.3 ± 7.1 years in LigaSure group. Our results are comparable to results of study by Baloch et al. from Karachi reported a mean age of 42.38 ± 17.39 years for patients undergoing open thyroidectomy for benign multinodular goiter [17-18].

In our study all surgeries were performed by the consultant surgeons with same level of experience. Both groups had similar demographics, comorbidity, diagnoses, and surgical procedure. We found that the use of LigaSure significantly reduced surgical time and hospital stay. We observed a statistically significant difference between the two groups in postoperative hypocalcemia and hematoma rates. Some other reports in literature have shown results similar to our study [19-20]. We observed two temporary RLN injuries in conventional group (1.9%) and one (0.9%) in LigaSure group. RLN injury was obvious because of hoarseness of voice and evaluated by IDL examination performed by the consultant ear, nose, and throat surgeon. There was no permanent RLN palsy in either group.

Transient hypocalcemia was seen in seven (6.8%) patients in conventional suture tie technique, whereas only three (2.9%) patients in LigaSure technique group. The difference was statistically significant (p<0.05). Similarly permanent hypoparathyroidism was also statistically significantly higher in conventional suture tie technique than LigaSure technique, 3.4% versus 0.9% respectively. Our results were comparable to results from literature. The change in technique with LigaSure facilitates dissection of the thyroid lobes and helps to reduce surgical time. They also claimed that a decreased requirement for lateral skin retraction and the reduction in incision length in the LigaSure group probably reduced the postoperative pain [20]. Some studies suggest that using LigaSure is more expensive than conventional suture ligation (CSL), but this may be reduced by using one device for several patients [21].

Our study reported a statistically significant difference in the mean operation time between the LigaSure group and conventional vascular ligature group (p<0.01). The mean operative time in LigaSure group was 92 ± 9.6 min while in the conventional suture tie technique group, the mean operation time was 123 ± 7.9 min. In their comparative study Ciftci showed difference in operative times (∼15%) between harmonic and traditional suture tie groups but this difference was not statistically significant. The shorter operative times associated with Harmonic Focus are likely due to its unique ultrasound-based technology, which enables simultaneous cutting and coagulation [22]. A meta-analysis by Zhang et al. also reported significant reduction in operative time in LigaSure group versus conventional suture ligation method (p<0.00001) [17, 23]. Studies have reported mean operative times ranging from 58 to 115.54 min for total thyroidectomy with LigaSure small jaw device and mean operative times between 75 and 153.45 min for conventional vascular ligature technique [17, 22-23]. In our study, despite advantage of decreased operative time the cost of surgery was much higher in LigaSure group because of the cost of the device. Although there is a significant decrease in operative time seen, the cost advantage is likely negated by the cost of the devices themselves [1].

Many researchers have reported results similar to our study. These studies demonstrated statistically significant (p<0.005) difference in intraoperative bleeding between LigaSure small jaw group and conventional technique group [17, 19, 22, 24]. In our study there was only one case of postoperative hematoma formation in the conventional vascular ligation group (0.9%) while none of the patients in the LigaSure group developed hematoma formation (p>0.05). In their retrospective analysis Dehal et al. reported an incidence (1.5%) of postoperative hematoma after thyroid surgery. Similar incidence has been reported in literature (0.1%-4.7%) [25]. In our study there was only one case of postoperative hematoma formation in the conventional vascular ligation group (0.9%) while none of the patients in the LigaSure group developed hematoma formation (p>0.05). Results from randomized control trial by Ramouz et al. showed there was no significant difference in postoperative hematoma formation between Ligasure small jaw and Harmonic scalpel groups. Four patients developed postoperative hematoma. Only one patient required reoperation to acquire hemostasis [26]. Ligasure small jaw is a bipolar energy based device that seals vessels by denaturation of collagen and elastin in vessels and adjacent connective tissue. It provides effective hemostasis by sealing tiny blood vessel up to 7 mm in the bed of thyroid gland. In conventional suture tie thyroidectomy these vessels continue to ooze because they cannot be ligated for control of bleeding [27-28]. Since LigaSure operates at a lower temperature (80°F) as compared to other EBDs like Harmonic Focus, it causes reduced thermal damage [26-27]. The extent of thermal injury with LigaSure has been reported as less than 1 mm to as wide as 3 mm. Despite variable heat dissipation these studies have reported similar results related to RLN injury [29]. Use of LigaSure in thyroid surgery significantly decreases risk of detrimental vascular and nerve damage. In comparison to conventional thyroidectomy, LigaSure vessel sealing system has been suggested to reduce operative time, RLN, and parathyroid injury [27]. 

## Conclusions

LigaSure was significantly advantageous over conventional technique in reducing operation time as well as perioperative and postoperative blood loss and parathyroid injury. However, RLN injuries were similar in both groups. The reduction of operative times resulted in decreased operating room occupancy costs but the overall cost of surgery was significantly higher in LigaSure group.
